# Histologically confirmed high-grade cervical intraepithelial neoplasia successfully treated with topical Paiteling: a two-case report

**DOI:** 10.3389/fmed.2025.1503083

**Published:** 2025-10-15

**Authors:** Jing Li, Jie Yang, Zhiying Yu, Songqing Guan

**Affiliations:** ^1^Department of Gynecology, Women’s Hospital, School of Medicine, Zhejiang University, Hangzhou, China; ^2^Shenzhen Key Laboratory of Reproductive Immunology for Peri-Implantation, Shenzhen Zhongshan Institute for Reproductive Medicine and Genetics, Shenzhen Zhongshan Obstetrics & Gynecology Hospital, Shenzhen, China; ^3^Department of Gynecology, Shenzhen Second People’s Hospital, the First Affiliated Hospital of Shenzhen University, Shenzhen, China

**Keywords:** high-grade cervical intraepithelial neoplasia (HSIL), topical Paiteling, human papillomavirus, fertility-preserving therapy

## Abstract

**Background:**

High-grade cervical intraepithelial neoplasia (HSIL) threatens fertility-aged women worldwide. Surgical excision remains standard but may compromise future pregnancy. Paiteling, a multi-herbal topical preparation, has shown *in vitro* anti-HPV activity, yet clinical evidence is scant.

**Case presentation:**

Two reproductive-aged women with biopsy-proven HSIL refused loop electrosurgical excision. Biopsy slides were re-reviewed independently with p16 and Ki-67 immunostaining. Case 1 was CIN II and Case 2 was CIN III. Both patients received topical Paiteling (0.5 mL/lesion, three times weekly) plus 1% vaginal douche for 10 weeks.

**Intervention & outcome:**

Lesions regressed completely within the treatment course. HPV-DNA testing confirmed HPV clearance by week 12. Follow-up colposcopy, cytology and directed biopsy remained negative at 12 months (Case 1) and 16 months (Case 2). Only mild transient vaginal burning occurred.

**Conclusion:**

Topical Paiteling achieved histological and virological remission in two pathologically confirmed HSIL cases that declined excisional surgery. The findings suggest a fertility-preserving option worth systematic evaluation in prospective trials.

## Introduction

Cervical intraepithelial neoplasia (CIN) is a precancerous lesion caused by persistent infection with high-risk human papillomavirus (HPV), closely linked to the progression to cervical cancer (1). CIN is classified into low-grade (LSIL or CIN I) and high-grade lesions (HSIL, encompassing CIN II and III) (2). Persistent infection with high-risk HPV subtypes such as HPV16 and 18 is a key risk factor for developing high-grade CIN and cervical cancer (2–4). Untreated high-grade CIN has a risk of progression to invasive cancer, with CIN II having a 18% progression rate and CIN III ranging from 22 to 40% within 30 years (3, 5, 6). Globally, the incidence of HSIL in reproductive-aged women continues to rise, reaching 498/100,000 in recent Chinese data (7). Given this risk, current WHO and ASCCP guidelines recommend surgical excision—most commonly cold knife conization (CKC), laser conization, or the loop electrosurgical excision procedure (LEEP)—as the standard treatment (8–10). Conization, depending on cone size, is also considered a fertility-sparing treatment, albeit with variable obstetric impact (10–12). Surgical interventions, though effective, come with risks such as bleeding, infection, and scarring (13), as well as potential complications like cervical insufficiency, stenosis, and preterm birth in women of reproductive age (14, 15). As fertility preservation becomes an increasing concern for women of reproductive age, there is a growing demand for conservative alternatives for high-grade CIN (14).

Non-surgical strategies—including photodynamic therapy, immunomodulatory agents, and Traditional Chinese Medicine (TCM)—are attracting interest for their potential to avoid cervical tissue loss (10, 16–18). Among TCM approaches, external therapies such as fumigation, rubbing, and soaking with plant-derived preparations have been reported to achieve local viral clearance without the tissue damage associated with surgery (16, 17). Paiteling, a pure TCM formulation, has shown encouraging results in treating HPV infection and condyloma acuminatum (17, 19–21), yet its role in managing high-grade CIN remains unexplored. Here, we present a retrospective review of two cases of reproductive-age women with biopsy-confirmed HSIL and concomitant vaginal and cervical condyloma acuminatum, successfully managed with topical Paiteling.

## Case presentation

### Case 1

**FIGURE 1:**
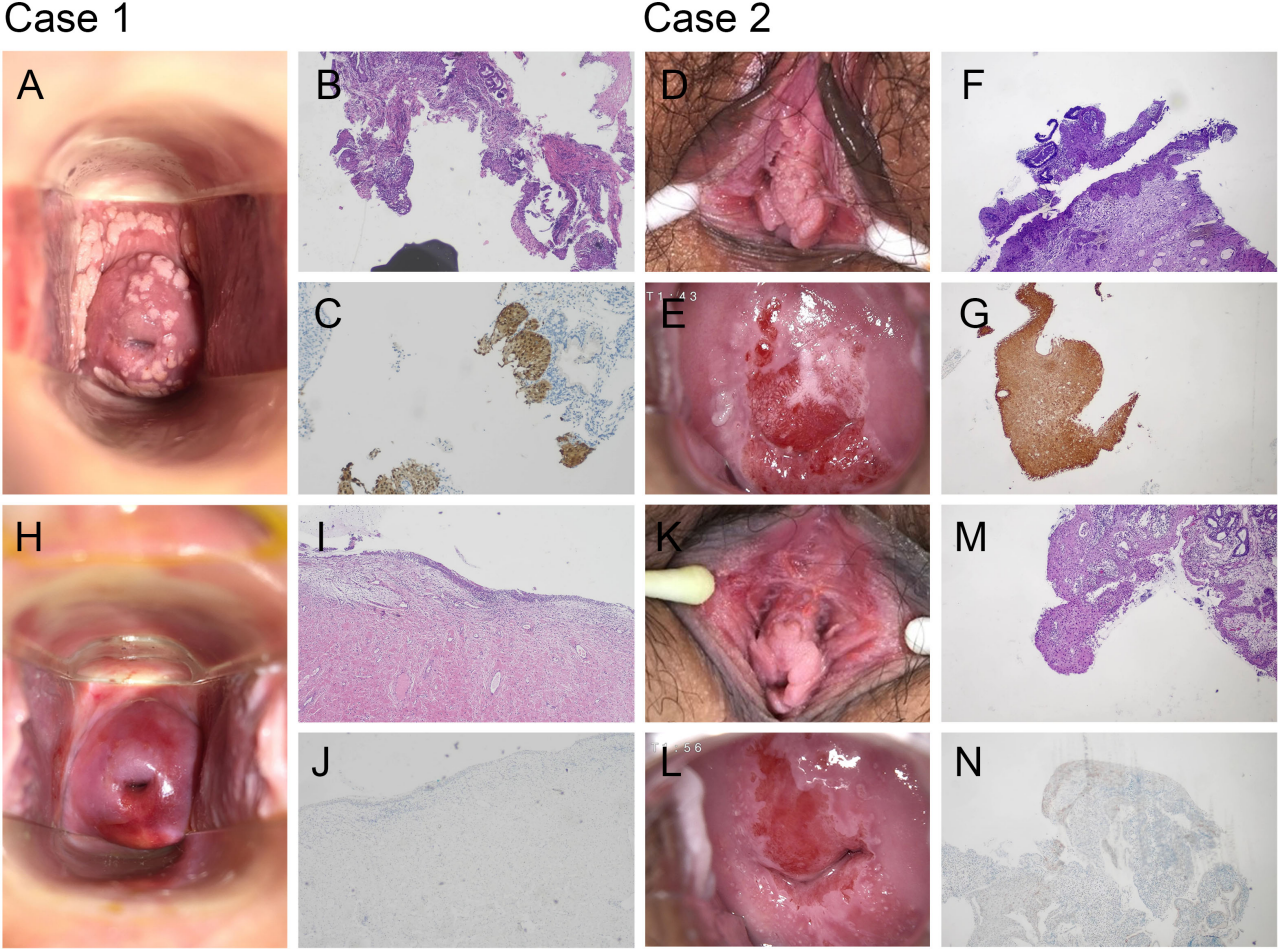
Topical Paiteling achieves complete regression of high-grade cervical intraepithelial neoplasia. **(A)** Case 1: Extensive condylomatous plaques on the vaginal wall and ectocervix before treatment. **(B,C)** Case 1: Baseline cervical biopsy confirming cervical intraepithelial neoplasia grade II (CIN II)/high-grade squamous intraepithelial lesions (HSIL). Hematoxylin and eosin (H&E) staining shows full-thickness atypia **(B)**. p16 immunostaining is diffusely positive **(C)**. **(D,E)** Case 2: Vulvar and cervical lesions at presentation. **(F,G)** Case 2: Baseline biopsy confirming CIN III/HSIL with diffuse p16 positivity. **(H)** Case 1: Macroscopic view after the 10-week course, showing smooth epithelium without visible lesions. **(I,J)** Case 1: Post-treatment biopsy reveals only chronic cervicitis and is p16-negative, consistent with histological remission. **(K,L)** Case 2: Vulva and cervix after therapy, demonstrating complete clinical resolution. **(M,N)** Case 2: Follow-up biopsy shows normal epithelium and absent p16 expression, indicating eradication of CIN III/HSIL. All histological images: original magnification ×100.

A 29-year-old nulliparous woman of Han ethnicity, residing in Shenzhen, China, was diagnosed with high-risk HPV-infected CIN II combined with VaIN I-II during a community cervical cancer screening and presented to our hospital’s gynecology department for further treatment in April 2018. Gynecological examination revealed multiple warty vegetations on the vaginal wall and cervix, characterized by papillary hyperplasia with dirty gray exudate on the surface, accompanied by an unpleasant odor (Figure 1A). A positive contact bleeding test was observed. The ThinPrep Cytology Test (TCT) revealed abnormal DNA ploidy cells, and cervical HPV typing confirmed HPV16 positivity (Figures 1B, C). Colposcopy and biopsy findings showed CIN II with condyloma on the cervix, and Vaginal intraepithelial neoplasia (VaIN) I-II with condyloma on the anterior and right side of the vaginal wall (Supplementary Table 1). Her medical history was unremarkable, with no history of chronic illness, immunodeficiency, prior cervical procedures, or prophylactic HPV vaccination. She denied any family history of malignancy or genetic cancer syndromes, and her psychosocial history revealed no occupational or behavioral risk factors for HPV persistence (Table 1).

**TABLE 1:** Baseline demographics, clinical course, HPV testing, and interventions for both cases.

Parameter	Case 1	Case 2
Age (years)	29	24
Ethnicity	Han	Han
Parity	0	0
Smoking status	Never-smoker	Never-smoker
Relevant comorbidities	None	None
Prior cervical procedures	None	None
HPV vaccination status	Not vaccinated	Not vaccinated
Family history of tumors	None	None
Main presenting concerns	Genital warts; abnormal cytology	Genital warts; abnormal cytology
Prior related interventions	None	None
Final histology	CIN II (HSIL), VaIN I-II	CIN II-III (HSIL)
p16	Diffuse+	Diffuse+
Ki-67	>1/3 epithelium	>2/3 epithelium
Baseline HPV	HPV16	HPV16, HPV52, HPV58
Interventions	10-week Paiteling treatment	10-week Paiteling treatment
HPV status at week 12	Negative	Negative
Follow-up (months)	12	16
Recurrence	None	None

HPV, human papillomavirus; CIN, cervical intraepithelial neoplasia; VAIN, vaginal intraepithelial neoplasia.

### Case 2

A 24-year-old nulliparous woman of Han ethnicity presented to the gynecology department in November 2021 seeking treatment advice after being diagnosed with high-risk HPV–infected CIN III. Her primary concerns were the presence of multiple genital warts and the recent histologic confirmation of HSIL. Gynecological examination revealed multiple hyperkeratotic, cauliflower-like warty lesions on the vulva and vagina, with a positive contact bleeding test (Figures 1D, E). Cervical HPV typing was positive for HPV16, 52, and 58 (Supplementary Table 1). Colposcopy and biopsy demonstrated CIN II–III with severe dysplasia and glandular involvement, along with a positive endocervical curettage (ECC) for CIN III (Figures 1F, G; Supplementary Table 1). The squamocolumnar junction and lesion margins were fully visualized on colposcopy. Her medical history included a recent high-risk HPV infection and a 1-year history of intermittent oral contraceptive use; she was a never-smoker and had no significant comorbidities, prior cervical interventions, or family history of cervical cancer. She had not received HPV vaccination prior to presentation. Psychosocial history was unremarkable for HPV persistence risk factors. She had not attempted other medical interventions for her current lesions before presentation (Table 1).

### Inclusion and exclusion criteria, diagnostic assessment, treatment and follow-up

The inclusion criteria for this case study were women aged 18–45 years, diagnosed with high-grade CIN (CIN II or III) and HPV-positive condyloma acuminatum, with no prior cervical surgery or radiation. Exclusion criteria included pregnancy or lactation, allergies to traditional Chinese medicine, immunocompromised conditions, and other malignancies or severe gynecological disorders. Diagnostic evaluation included pelvic examination, ThinPrep Cytology Test (TCT), high-risk HPV genotyping, colposcopy with directed cervical and vaginal biopsies, and endocervical curettage (ECC) where indicated. Histopathological assessment was performed by two independent senior pathologists. The principal diagnostic challenge was differentiating high-grade CIN from reactive epithelial changes caused by extensive condylomatous lesions, which could obscure lesion margins during colposcopic evaluation. Differential diagnoses considered included low-grade squamous intraepithelial lesions (LSIL), benign condyloma acuminatum without intraepithelial neoplasia, and vaginal intraepithelial neoplasia (VaIN) without concurrent cervical involvement. The final diagnosis in both cases was established based on concordant histology from multiple biopsy sites and positive hr-HPV testing. Follow-up was conducted at 1, 6, and 12 months post-treatment. At each visit, HPV testing was performed to assess for persistent infection, and TCT along with colposcopy were used to monitor for recurrence. If request by patients or abnormal findings were observed, a cervical biopsy was conducted at the 12-month visit. Patients were also asked to report any abnormal symptoms, and further tests were done if necessary.

Following the WHO and ASCCP guidelines, our senior gynecologists initially recommended LEEP surgery (8, 9). A detailed counseling session was conducted, during which the patients were thoroughly informed about the benefits of surgical treatment, as well as the risks of recurrence, scarring, cancerization, and obstetric complications, such as cervical insufficiency and preterm birth. These discussions were fully documented in the patient records. Both patients, after considering these risks, declined surgery, expressing concerns over potential long-term effects. This refusal of surgery was formally recorded, with the patients signing a document acknowledging their decision to decline excisional therapy and the associated oncologic risks. Non-surgical alternatives, including photodynamic therapy and Traditional Chinese Medicine (TCM), were then presented and discussed in terms of their safety, efficacy, and potential for fertility preservation. After thorough deliberation, both patients opted for the standard 10-week Paiteling treatment regimen, consisting of 6 weeks of cervical application followed by 4 weeks of vaginal douching under physician supervision (Supplementary Table 2). Treatment adherence was complete for both cases. Adverse events were monitored throughout therapy and documented according to the Common Terminology Criteria for Adverse Events (CTCAE, version 5.0) framework (22) where applicable. Symptom severity, including intermittent mild pain and a burning sensation, was assessed qualitatively via patient self-report; no events met the criteria for Grade ≥ 2 severity. All symptoms were transient, resolved spontaneously without medical intervention, and did not lead to treatment interruption or dose modification. Upon completion of the regimen, both patients expressed satisfaction with the outcomes. Follow-up evaluations demonstrated clinical remission, negative hr-HPV testing, and no abnormal findings on TCT, colposcopy, or cervical biopsy (Case 1: Figures 1H–J; Case 2: Figures 1K–N) (Table 1; Supplementary Table 1).

## Discussion

High-grade cervical intraepithelial neoplasia (CIN II–III, HSIL) carries a low likelihood of spontaneous regression and a lifetime risk of progression to invasive cancer of 22%–40% (3, 5, 6). Accordingly, both the WHO and ASCCP recommend excisional treatment as the gold standard (8, 9). Nevertheless, excisional procedures may be followed by persistent high-risk human papillomavirus (hr-HPV) infection in up to 68% of patients, with ongoing viral persistence markedly increasing recurrence risk (4, 23). Furthermore, meta-analyses have demonstrated that prior excision is associated with significantly elevated risks of adverse obstetric outcomes, including a 1.78–3.78-fold increase in preterm birth, a 1.99-fold increase in premature rupture of membranes, and a 1.94-fold increase in low birth weight infants compared with women without prior excision (11, 14, 24).

Medical and other non-excisional approaches, such as topical immunomodulators (e.g., imiquimod), photodynamic therapy, and Traditional Chinese Medicine formulations, may avoid cervical tissue loss and thus potentially mitigate these obstetric risks (10, 16–18). Topical imiquimod and photodynamic therapy, which have shown lesion regression rates of 50%–80% in selected patients (25, 26). The APL-1702 (a photodynamic therapy drug-device combination) phase III trial also reported favorable HPV clearance and safety outcomes in a large HSIL cohort (27). Emerging robotic-assisted precision excision techniques also aim to minimize tissue removal while ensuring complete lesion eradication, aligning with the principles of non-excisional therapies that prioritize fertility preservation and reduction of obstetric complications (28, 29). In addition, adjuvant HPV vaccination has been shown to significantly lower the recurrence of high-grade CIN, with odds ratios of 0.33 for CIN II + and 0.28 for CIN III, underscoring its potential role in secondary prevention (30). Compared with these modalities, Paiteling represents a non-invasive, cost-effective alternative with potential antiviral and immunomodulatory effects, and without the need for specialized equipment. Our report suggests that Paiteling may offer comparable histological and virological remission, with minimal systemic adverse effects. However, the oncologic safety of non-excisional strategies remains less well established, with limited high-quality evidence on long-term recurrence rates, particularly in HSIL. Therefore, both oncologic safety and obstetric outcomes must be weighed when comparing medical and excisional approaches. Treatment selection should be individualized, balancing reproductive goals against the need to minimize cancer progression risk, ideally within the framework of shared decision-making and close post-treatment surveillance.

Paiteling, a topical formula developed by the Chinese Academy of Medical Sciences, combines *Brucea javanica*, *Isatis tinctoria*, *Hedyotis diffusa*, *Sophora flavescens*, and *Cnidium monnieri* (20, 21) (Supplementary Table 2). The alkaloids, flavonoids and quassinoids in these herbs synergistically lyse infected epithelial cells; shed necrotic debris removes latent HPV, while sea-buckthorn oil constituents (anthocyanins, quercetin) dampen inflammation and support re-epithelialisation (31). Paiteling down-regulates E6/E7 transcription, blocks PI3K/Akt signaling, and triggers apoptosis in cervical cancer lines (21, 32).

Clinical evidence—although limited to low-grade disease—has been encouraging. Cure rates of 92%–100% for condyloma acuminatum and 89% for LSIL have been reported, with minimal pain and no scarring (17, 19–21, 33). Our two HSIL cases extend these findings: topical Paiteling cleared hr-HPV and achieved durable histological remission without compromising cervical architecture. To the best of our knowledge, this is the first report on the use of Paiteling in biopsy-confirmed HSIL coexisting with genital warts. These preliminary observations are encouraging but warrant cautious interpretation. The study is inherently constrained by its retrospective nature, two-case sample size, single-center setting, absence of a comparator group, and relatively limited follow-up duration, which together limit the generalizability of the findings. Obstetric outcomes were not evaluated, and any conclusions regarding safety remain provisional. The evaluation of lesion regression in this report relied on histopathological clearance and HPV negativity. Future studies should incorporate standardized, validated outcome scoring systems for treatment response—such as the Lower Anogenital Squamous Terminology (LAST) criteria or immunologic markers to enhance objectivity and clinical translatability. Given the 12- to 16-month follow-up, our study cannot determine long-term recurrence or progression risk, particularly as latent HPV reactivation may occur beyond the follow-up horizon. Accordingly, our findings should be interpreted as preliminary, and long-term surveillance is essential for drawing definitive conclusions about sustained remission. No systemic side effects were reported in our cases; however, detailed safety data, particularly regarding systemic absorption, endocrine disruption, or reproductive toxicity of Paiteling, are lacking. Preclinical pharmacokinetic studies and reproductive toxicity models are warranted before wider clinical adoption, particularly in women seeking future pregnancies. Rigorous, randomized controlled trials incorporating virological endpoints and long-term obstetric follow-up are required to establish the efficacy, optimal regimen, and safety profile of this approach in reproductive-age women who decline or are ineligible for excisional therapy. Furthermore, optimal management of CIN should be grounded in comprehensive patient counseling, formal documentation of informed consent, and ethics committee oversight, ensuring that patients are fully apprised of the potential risks and benefits of all therapeutic options, and that autonomy is upheld throughout the decision-making process.

## Conclusion

Topical Paiteling may be a promissing non-excisional option for selected women with HSIL who prioritize fertility preservation. Rigorous clinical trials are required to establish efficacy, safety, and obstetric impact.

## Data Availability

The original contributions presented in this study are included in this article/Supplementary material, further inquiries can be directed to the corresponding authors.
