# Supplementary material for: Histologically confirmed high-grade cervical intraepithelial neoplasia successfully treated with topical Paiteling: a two-case report
Source: Front Med (Lausanne). 2025 Oct 15;12:1503083. doi: 10.3389/fmed.2025.1503083 (PMC12568692; doi:10.3389/fmed.2025.1503083)
Supplement: Supplementary file 1 [file Table_1.docx]

**Table S1. Timeline of pre- and post-treatment HPV testing, ECC, colposcopy and cervical biopsy pathology results in the two cases.**

| **Stage** | **Case 1** | **Case 2** |
| --- | --- | --- |
| **Pre-treatment** | **2018/04/13 (Pre-treatment)**  HPV-DNA genotyping：HPV16 (+); TCT：abnormal DNA ploidy cells (+)，atypical squamous cells (+); Colposcopy: squamocolumnar junction is not visible，transformation zone CIN III；Cervical Biopsy Pathology: (Cervical points 2 and 7) CIN II with condyloma，（Anterior and right walls of vagina）VAIN I -II with condyloma | **2021/11/29 (Pre-treatment)**  HPV-DNA genotyping：HPV16/52/58 (+); ECC：Free squamous epithelium showing high-grade squamous intraepithelial lesion (HSIL), CIN III, with focal condyloma; Cervical Biopsy Pathology: Local squamous epithelial hyperplasia at cervix points 1 and 8；Immunohistochemistry (IHC): (Cervix point 1) P16 (+), Ki67 (60%+), focal HSIL (CIN II-III, invading the gland); (Cervix point 8) P16 (-), Ki67 (positive in sub-basal layer), chronic inflammation. |
| **Post-treatment** | **2018/08/24 (Post-standard treatment)**  HPV-DNA genotyping: HPV (-); TCT：No abnormal DNA ploidy cells. | **2022/02/18 (Post-standard treatment)**  HPV-DNA genotyping: HPV16/52/58 (+); ECC: chronic inflammation; Cervical Biopsy Pathology and IHC：(Cervical points 1, 6, 8, and 12) P16 (-), Ki-67 (positive in basal cells), chronic cervical inflammation presenting with condyloma-like changes, with active glandular epithelial hyperplasia |
|  | **2019/09/08 (12 months post-treatment)**  HPV-DNA genotyping: HPV (-); ECC: (Endocervical canal) Mucous and inflammatory exudate with abundant keratinized squamous epithelium, a small amount of well-differentiated glandular epithelium, with no other pathological changes noted. | **2023/03/16 (12 months post-treatment)**  HPV-DNA genotyping: HPV (-); ECC: (Endocervical canal) Mucous and inflammatory exudate with abundant completely keratinized squamous epithelium, a small amount of well-differentiated glandular epithelium, and a large quantity of neutrophils, with no other pathological changes noted;  2023/03/16 Cervical Biopsy Pathology and IHC: (Cervical point 11) P16(-), Ki-67(about 60%+). Cervical tissues showed acute inflammatory changes. Partial area showed superficial granulation tissue changes with some fragmented squamous epithelium, most of the areas are normal. The microscopic morphology was consistent with post-treatment response, suggesting a possibility of repair. |
|  |  | **2023/07/16 (16 months post-treatment)**  HPV-DNA genotyping: HPV(-); TCT(-); Colposcopy: No abnormalities detected in the cervix. |

Abbreviations: HPV, Human papillomavirus; ECC, Endocervical curettage; TCT, ThinPrep Cytology Test; CIN, Cervical intraepithelial neoplasia; VAIN, Vaginal intraepithelial neoplasia.

**Table S2. Effective ingredients and standard treatment plan of paiteling***

| **Effective Ingredients** | **Functions** |
| --- | --- |
| Cnidium Monnieri | Has anti-allergic and antipruritic properties, and prevents skin lesions. |
| Brucea javanica | Contains a variety of alkaloids, bitter principles, and oleic acids, which are effective against tumors, inflammation, and skin exfoliation. Has a strong inhibitory effect against HPV virus. |
| Sophora flavescens | Contains sophoracarpin, which acts as an antipyretic, antifungal, and antiviral agent. |
| Hedyotis diffusa | Has anti-tumor and anti-inflammatory properties. |
| Folium Isatidis | Contains indigo, brassin, and glycoside compounds, which provide heat-clearing, detoxifying, broad-spectrum antibacterial, and antiviral effects. |
| Sea buckthorn oil | Anti-inflammatory, immune-enhancing, stimulates cell regeneration and promotes epithelial tissue healing. As part of external treatment, it is used to promote tissue repair. |
| Standard Treatment Plan | Stage 1 (Elimination of HPV virus and lesions): Each treatment lasts for 2 hours, conductes 3 times a week for 6 consecutive weeks. During each treatment, a cotton swab soaked in 5ml of paiteling solution is applied internally and externally to the cervix. After 2 hours, removes the cotton swab to complete the treatment. Following 3 consecutive days of using paiteling, switch to Sea buckthorn oil treatment (provided within the product) for 4 days of repair, alternating treatment for 6 weeks. |
|  | Stage 2 (Prevention of recurrence): Use a 1:50 dilution of paiteling solution to douche the vagina once a day for 4 consecutive weeks. |

* Paiteling is provided by Beijing Paite Boen Biotechnology Development Group Co., Ltd. (Beijing, China).
